# A monoclonal antibody raised against human EZH2 cross-reacts with the RNA-binding protein SAFB

**DOI:** 10.1242/bio.059955

**Published:** 2023-06-07

**Authors:** Rachel E. Cherney, Christine A. Mills, Laura E. Herring, Aki K. Braceros, J. Mauro Calabrese

**Affiliations:** ^1^ Department of Pharmacology, University of North Carolina at Chapel Hill, 120 Mason Farm Road, Chapel Hill, NC 27599, USA.; ^2^ RNA Discovery Center, University of North Carolina at Chapel Hill, 120 Mason Farm Road, Chapel Hill, NC 27599, USA.; ^3^ Lineberger Comprehensive Cancer Center, University of North Carolina at Chapel Hill, 120 Mason Farm Road, Chapel Hill, NC 27599, USA.; ^4^ Curriculum in Genetics and Molecular Biology, University of North Carolina at Chapel Hill, 120 Mason Farm Road, Chapel Hill, NC 27599, USA.; ^5^ Proteomics Core Facility, University of North Carolina at Chapel Hill, 120 Mason Farm Road, Chapel Hill, NC 27599, USA.; ^6^ Curriculum in Biochemistry and Biophysics, University of North Carolina at Chapel Hill, 120 Mason Farm Road, Chapel Hill, NC 27599, USA.; ^7^ Curriculum in Mechanistic, Interdisciplinary Studies of Biological Systems, University of North Carolina at Chapel Hill, 120 Mason Farm Road, Chapel Hill, NC 27599, USA.

**Keywords:** EZH2, PRC2, Polycomb, SAFB, Xist, LncRNA

## Abstract

The Polycomb Repressive Complex 2 (PRC2) is a conserved enzyme that tri-methylates Lysine 27 on Histone 3 (H3K27me3) to promote gene silencing. PRC2 is remarkably responsive to the expression of certain long noncoding RNAs (lncRNAs). In the most notable example, PRC2 is recruited to the X-chromosome shortly after expression of the lncRNA *Xist* begins during X-chromosome inactivation. However, the mechanisms by which lncRNAs recruit PRC2 to chromatin are not yet clear. We report that a broadly used rabbit monoclonal antibody raised against human EZH2, a catalytic subunit of PRC2, cross-reacts with an RNA-binding protein called Scaffold Attachment Factor B (SAFB) in mouse embryonic stem cells (ESCs) under buffer conditions that are commonly used for chromatin immunoprecipitation (ChIP). Knockout of EZH2 in ESCs demonstrated that the antibody is specific for EZH2 by western blot (no cross-reactivity). Likewise, comparison to previously published datasets confirmed that the antibody recovers PRC2-bound sites by ChIP-Seq. However, RNA-IP from formaldehyde-crosslinked ESCs using ChIP wash conditions recovers distinct peaks of RNA association that co-localize with peaks of SAFB and whose enrichment disappears upon knockout of SAFB but not EZH2. IP and mass spectrometry-based proteomics in wild-type and EZH2 knockout ESCs confirm that the EZH2 antibody recovers SAFB in an EZH2-independent manner. Our data highlight the importance of orthogonal assays when studying interactions between chromatin-modifying enzymes and RNA.

## Teaser

**Summary:** We report that a broadly used rabbit monoclonal antibody raised against human EZH2 cross-reacts with Scaffold Attachment Factor B in mouse embryonic stem cells under buffer conditions that are commonly used for chromatin immunoprecipitation.

## INTRODUCTION

The Polycomb Repressive Complex 2 (PRC2) is a conserved histone-modifying enzyme that represses gene expression by catalyzing mono-, di-, and tri-methylation of Lysine-27 on Histone H3 (H3K27me3). PRC2 contains invariant subunits, including SUZ12, EED, and a catalytic engine−either EZH1 or EZH2−as well as several auxiliary factors, which together serve to recruit PRC2 to specific genomic regions and modulate its catalytic activity. PRC2 is essential for mammalian development and is mutated in cancers and congenital disorders, underscoring its importance in gene regulation (Laugesen et al., 2016; Deevy and Bracken, 2019; Yu et al., 2019).

PRC2 is highly expressed during early development, where it responds in a dramatic fashion to specific long noncoding RNAs (lncRNAs; Schumacher et al., 1996; O'Carroll et al., 2001; Wang et al., 2001; Silva et al., 2003). Within hours after the *Xist* lncRNA becomes expressed in the embryo proper (or in mouse embryonic stem cells; ESCs), PRC2 is recruited over the span of the 165 megabase (Mb) X chromosome (Plath et al., 2003; Okamoto et al., 2004; Zylicz et al., 2019). Expression of *Xist* transgenes from autosomal locations likewise result in chromosome-wide recruitment of PRC2, supporting the view that the *Xist* lncRNA itself, and not merely the act of its transcription, recruits PRC2 to chromatin (Trotman et al., 2021).

Exactly how PRC2 is recruited to chromatin by lncRNAs such as *Xist* remains unclear. While investigating this topic, we discovered that a widely used antibody raised against human EZH2 reproducibly cross-reacts with an RNA-binding protein called Scaffold Attachment Factor B (SAFB) in mouse ESCs during immunoprecipitations (IPs) but not by western blot. SAFB is a chromatin-associated RNA-binding protein that has been implicated in transcriptional repression and associates with the lncRNA *Xist* and may yet prove to play important roles in modulating PRC2 function (Townson et al., 2004; Mukhopadhyay et al., 2014; Chu et al., 2015; Bousard et al., 2019; Huo et al., 2020; McCarthy et al., 2021; Yu et al., 2021). Nevertheless, the data presented below highlight the importance of orthogonal assays in studying interactions between histone-modifying enzymes and RNA.

## RESULTS AND DISCUSSION

By cross-linking immunoprecipitation (CLIP), PRC2 has been shown to associate with many nascent RNAs, a process that in certain cases may antagonize its enzymatic activity (Davidovich et al., 2013; Kaneko et al., 2013; Cifuentes-Rojas et al., 2014; Kaneko et al., 2014; Davidovich et al., 2015; Beltran et al., 2016; Wang et al., 2017; Beltran et al., 2019; Garland et al., 2019; Zhang et al., 2019; Long et al., 2020; Rosenberg et al., 2021). However, expression of the lncRNA *Xist* causes the near-immediate recruitment of PRC2 over the span of the inactive X-chromosome (Zylicz et al., 2019). Based on these data, we hypothesized that lncRNAs such as *Xist* recruit PRC2 indirectly, through RNA-binding proteins. Indeed, HNRNPK is an RNA-binding protein that appears to bridge PRC1 and *Xist*, providing precedent for a protein-bridging model for PRC2 (Pintacuda et al., 2017).

**Fig. 1.**
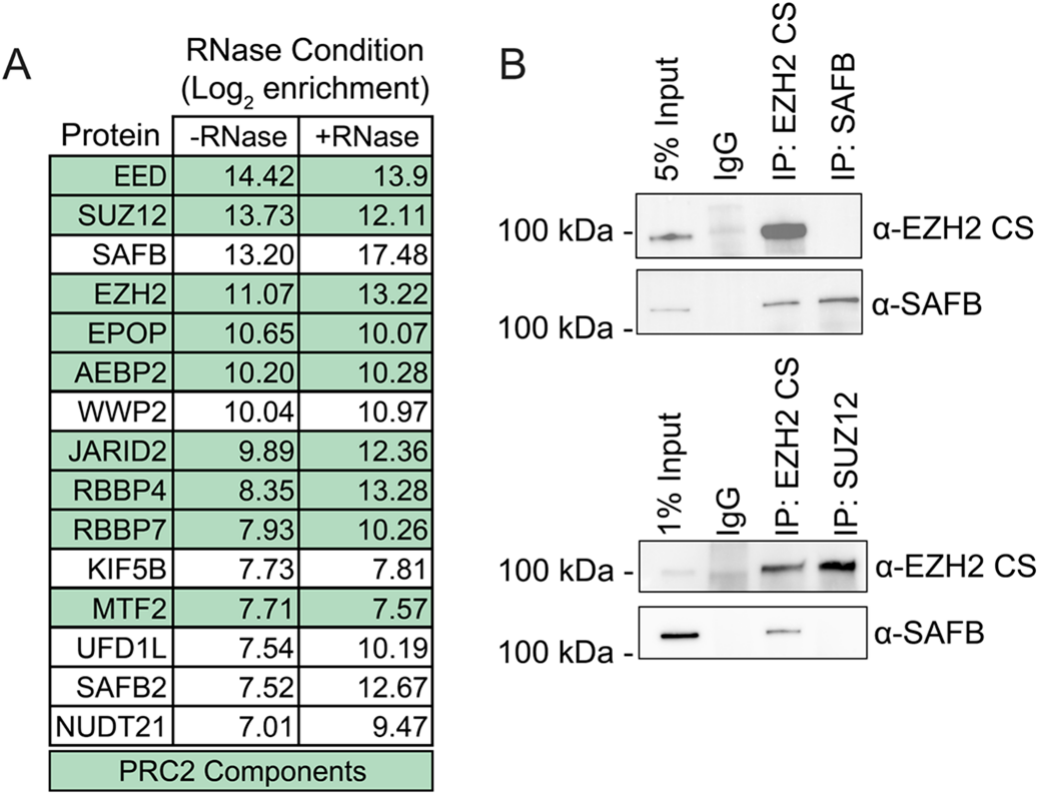
**IP-MS with EZH2-CS5246 identifies PRC2 complex members and SAFB.** (A) Log2 enrichment of top 15 proteins identified via IP-MS with EZH2-CS5246 in samples treated with or without RNase. PRC2 components are highlighted in green. IP-MS data can be found in Table S1. (B) IP-western analysis using EZH2-CS5246, SAFB, and SUZ12 antibodies.

To determine whether PRC2 associates with RNA-binding proteins, we performed mass spectrometry-based proteomics (IP- MS) for EZH2 using a rabbit monoclonal antibody raised against human EZH2 from Cell Signaling Technology (CS5246). We prepared nuclear extracts from ESCs and performed IP-MS using EZH2-CS5246 under conditions in which the extracts were and were not pre-treated with RNase. Ethidium bromide staining of RNA prepared from these nuclear extracts confirmed degradation of RNA in the RNase treated samples (not shown). We identified all PRC2 major subunits, as well as many of its known accessory factors, and did not detect peptides for our most-enriched proteins in IgG control, which led us to infer that the antibody and our IP approach were successful (Fig. 1A; Table S1). Notably, IP with EZH2-CS5246 identified several RNA-binding proteins under both RNase-free and RNase-treated conditions. The most highly-ranked of these was SAFB, which has previously been found to interact with EZH2 in human and mouse cells (Gao et al., 2012; Cao et al., 2014; Oksuz et al., 2018) and has been proposed to be necessary for H3K27me3 deposition at certain androgen-repressed genes (Mukhopadhyay et al., 2014). SAFB has also been shown to interact with *Xist* in mouse and human ESCs (Chu et al., 2015; Bousard et al., 2019; Yu et al., 2021). Using CS5246, we verified the interaction between EZH2 and SAFB via IP-western (Fig. 1B). EZH2-CS5246 IP retrieved SAFB, yet SAFB IP retrieved trace amounts of EZH2; likewise, IP with another core component of PRC2, SUZ12, did not retrieve SAFB (Fig. 1B). Nevertheless, the prior connections between SAFB, EZH2, and *Xist* were intriguing enough that we elected to study the interaction further.

**Fig. 2.**
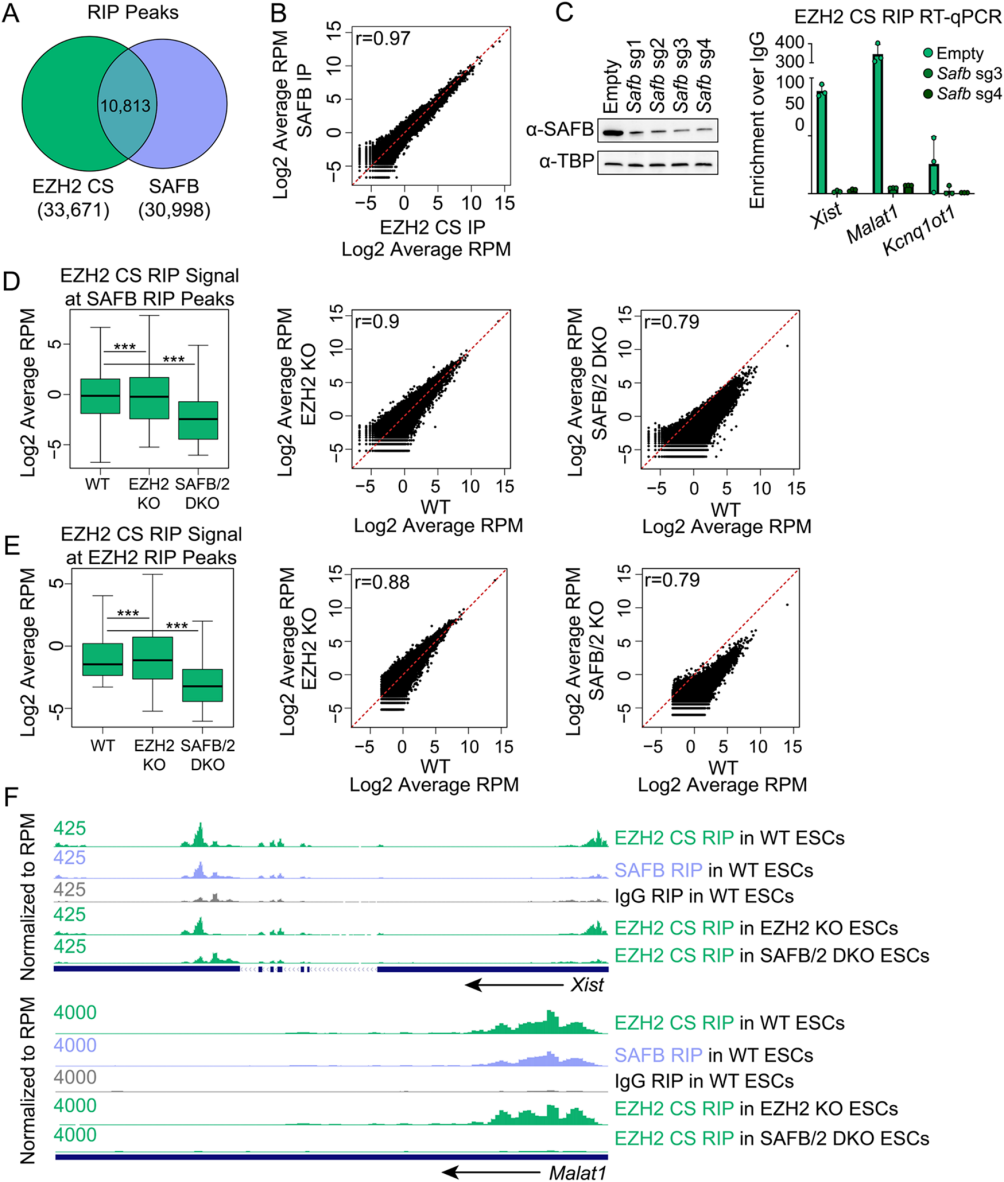
**EZH2-CS5246 recovers SAFB-dependent signal in RIP.** (A) Locational overlap between EZH2-CS5246 and SAFB RIP peaks. Hypergeometric distribution *P*-value, <2e-16. EZH2 RIP peaks can be found in Table S2. (B) SAFB and EZH2-CS5246 RIP signal under EZH2-CS5246 RIP peaks. Pearson's r=0.97. (C) Western blot showing SAFB levels following 3[Supplementary-material sup1]days of Cas9 induction. RT-qPCR detecting *Xist*, *Malat1*, and *Kcnq1ot1* in EZH2-CS5246 RIP from ESCs stably expressing a doxycycline-inducible Cas9 and no sgRNA (Empty) or one of two separate sgRNAs targeting SAFB. (D) EZH2-CS5246 RIP signal under SAFB RIP peaks in WT, EZH2 KO and SAFB/2 DKO cell lines. *P*-value, ***=<2e-16, *t*-test. (E) EZH2-CS5246 RIP signal under EZH2 RIP peaks in WT, EZH2 KO and SAFB/2 DKO cell lines. *P*-value, ***=<2e-16, *t*-test. (F) UCSC wiggle tracks of EZH2 and SAFB RIP signal in WT, EZH2 KO and SAFB/2 DKO ESCs over the lncRNAs *Xist* and *Malat1*.

While CLIP data show that PRC2 directly binds RNA somewhat promiscuously *in vivo*, we reasoned that specific, bridged interactions between RNA, RNA-binding proteins, and PRC2 could be missed by CLIP, which was designed to detect direct RNA/protein interactions. In contrast, bridged interactions might be apparent in RNA-IP (RIPs) from formaldehyde-crosslinked cells, which, in addition to direct interactions, can also reveal indirect ones in the form of RNA bound to proteins bound to the protein-target of the IP (Hoffman et al., 2015). To identify the RNAs retrieved by EZH2-CS5246 *in vivo*, we used a formaldehyde-based RIP-Seq protocol (Schertzer et al., 2019a), which relies on a series of washes that are also used in many chromatin immunoprecipitation (ChIP) protocols, including: one 5-min wash in a buffer that contains 500 mM NaCl, 1% Triton X-100, 0.1% sodium deoxycholate, and 0.1% SDS; and another 5-min wash in a buffer that contains 250 mM LiCl, 0.5% sodium deoxycholate, and 0.5% NP-40. Both wash conditions are predicted to destabilize non-specific protein-protein interactions. In parallel, we used the same RIP protocol to retrieve RNA associated with an endogenous SAFB antibody and mouse IgG as a negative control. By RIP-Seq, we observed a striking co-localization between the RNA retrieved EZH2-CS5246 and SAFB. Thirty-two percent of all EZH2 RIP peaks overlapped with SAFB RIP peaks (10,813/33,671 EZH2 peaks; *P*<2e-16 hypergeometric test; Fig. 2A; Table S2). When examining the top 10,000 EZH2 RIP peaks (ranked by EZH2 RIP signal), the overlap increased to 80% (7,968/10,000 EZH2 peaks; *P*<2e-16 hypergeometric test). Concordantly, SAFB and EZH2 RIP signal under EZH2 RIP peaks were highly correlated (Fig. 2B). Because transient depletion of SAFB by inducible CRISPR resulted in loss of EZH2-CS5246 signal at a subset of target lncRNAs (Fig. 2C), we elected to knockout SAFB and its paralogue SAFB2 in ESCs. Generation and study of SAFB/2 double-knockout (DKO) ESCs are described in greater depth in Cherney et al. (2022 preprint). We performed RIP-Seq using EZH2-CS5246 in wild-type and SAFB/2 DKO ESCs and observed a loss of signal at essentially all SAFB- and EZH2-CS5246-enriched regions in SAFB/2 DKO ESCs, indicating that RIP with EZH2-CS5246 retrieves RNA in a SAFB-dependent manner (Fig. 2D-F).

**Fig. 3.**
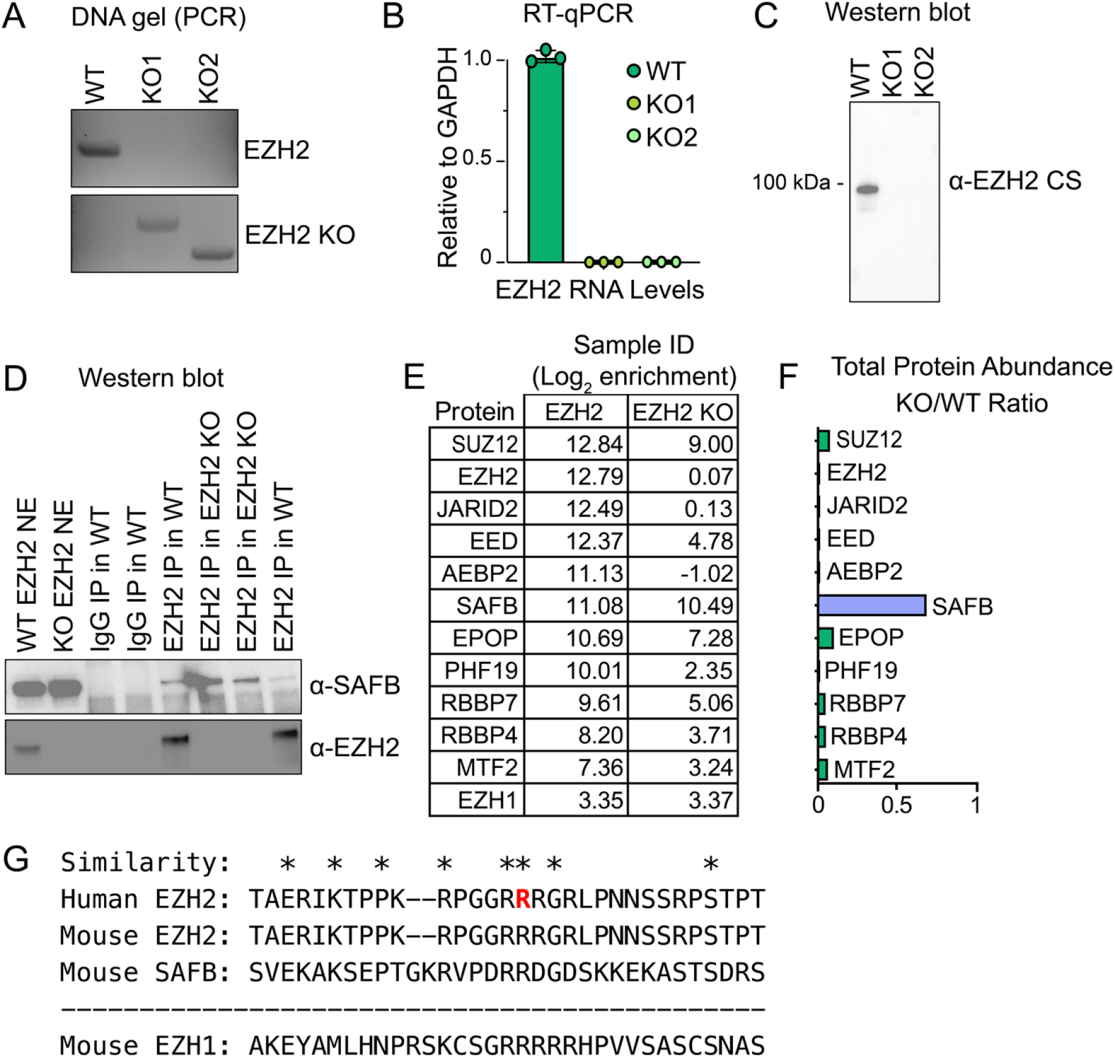
**EZH2-CS5246 pulls down SAFB in EZH2 KO ESCs.** (A) PCR validation of EZH2 KO. (B) RT-qPCR of EZH2 RNA levels in WT and two independent EZH2 KO clones. (C) Western blot using EZH2-CS5246 in WT and two independent EZH2 KO clones. (D) IP-western from WT and EZH2 KO nuclear extracts using EZH2-CS5246 or IgG control. (E) Log2 enrichment of PRC2 members and SAFB in EZH2-CS5246 IP-MS from WT and EZH2 KO nuclear extracts. IP-MS protein lists and quantification can be found in Table S1. (F) Ratio of protein abundance between WT and EZH2 KO nuclear extracts of PRC2 members and SAFB. (G) Sequence alignment of human EZH2-CS5246 epitope region, the analogous region from mouse EZH2, a predicted region of similarity in mouse SAFB, and mouse EZH1. The EZH2-CS5246 epitope surrounds Arg354 of human EZH2 and is denoted in red.

However, we remained concerned that previously, we did not retrieve SAFB in SUZ12 IPs, nor did we reproducibly retrieve EZH2 by SAFB IP (Fig. 1B). For this reason, we used CRISPR to generate EZH2 knockout (KO) ESCs. By PCR, RT-PCR, and western blot with EZH2-CS5246 we observed complete loss of EZH2 DNA, RNA, and EZH2 protein in EZH2 KO ESCs and no evidence of cross-reactivity with other proteins in the EZH2-CS5246 western blot (Fig. 3A-C). Nonetheless, RIP-Seq using EZH2-CS5246 showed essentially no change in signal at EZH2-CS5246-enriched regions in wild-type versus EZH2 KO ESCs (Fig. 2E and F; Table S2). Moreover, by IP-western and IP-MS, EZH2-CS5246 still retrieved SAFB even in EZH2 KO ESCs (Fig. 3D-F; Table S1). We conclude that under our RIP conditions, but not in western blots, EZH2-CS5246 cross-reacts with SAFB, and that by RIP, the RNA retrieved by EZH2-CS5246 requires the presence of SAFB and not EZH2.

We extracted the approximate epitope used to generate EZH2-CS5246 and aligned it to SAFB and observed no significant linear sequence similarity between the epitope and SAFB. However, we note that the approximate EZH2-CS5246 epitope does harbor several arginine and glycine residues, and regions of SAFB are also enriched in R/G residues, providing a possible reason for the cross-reactivity of EZH2-CS5246 with SAFB (Fig. 3G).

We also noted some similarity between the approximate epitope used to generate EZH2-CS5246 and EZH1 (Fig. 3G). The enrichment of EZH1 over IgG control does not change in IP-MS performed in wild-type versus EZH2 KO ESCs, consistent with the possibility of low-level cross-reactivity with EZH1 (Fig. 3E). However, in wild-type ESCs, the enrichment of EZH1 over IgG control is 500-fold lower than that of EZH2 and 200-fold lower than that of SAFB (Fig. 3E). Likewise, in EZH2-CS5246 IPs from wild-type cells, EZH1 is 1000-fold less abundant than either EZH2 or SAFB (Table S1). Thus, in ESC IPs with EZH2-CS5246, EZH2 is far more abundant than EZH1, and the extent of possible cross-reactivity between EZH2-CS5246 and EZH1 does not seem robust enough to account for the EZH2-independence of RNA retrieved by RIP.

**Fig. 4.**
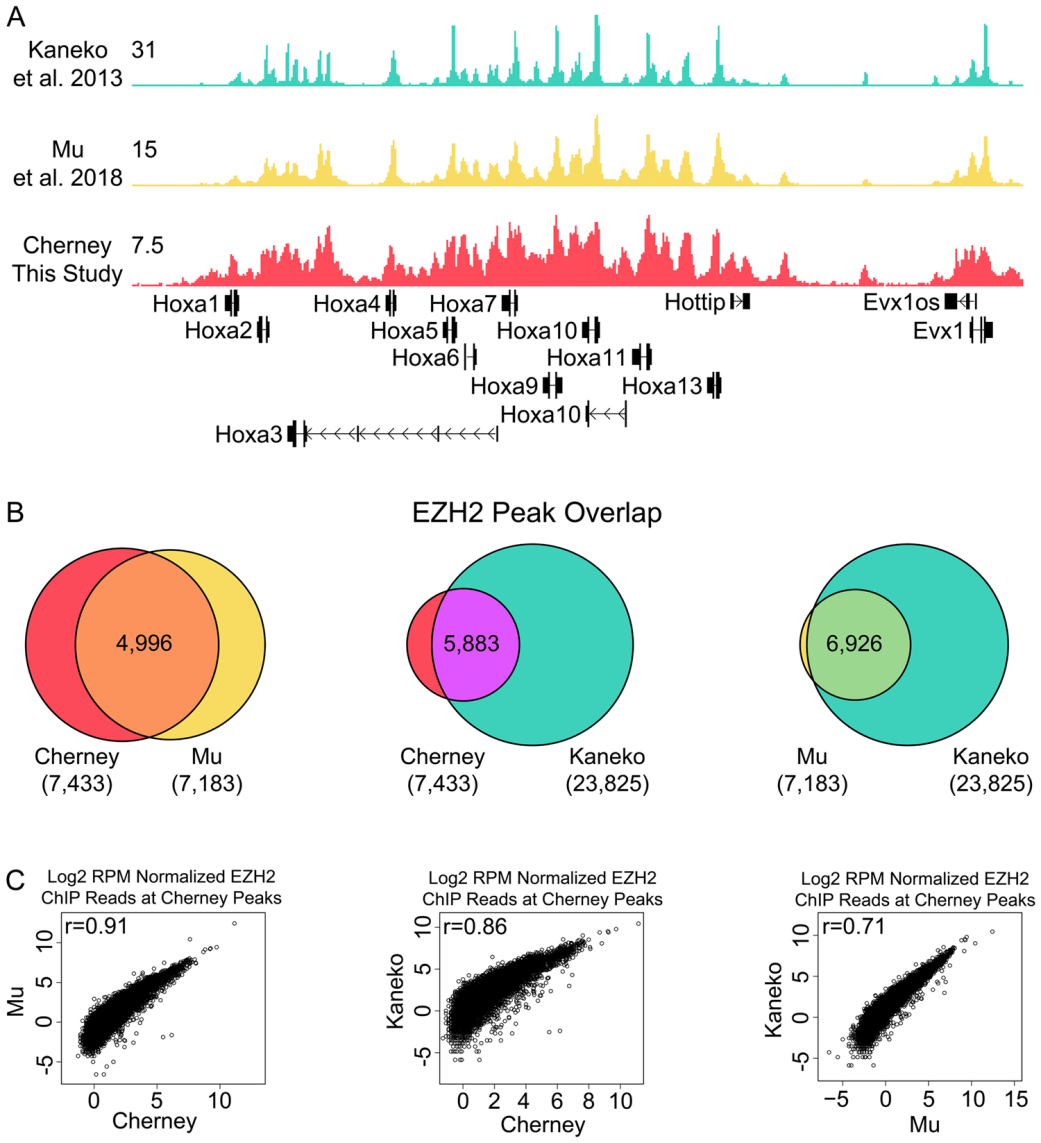
**Correlations between EZH2 ChIP-Seq data sets.** (A) EZH2 ChIP-Seq UCSC wiggle tracks of HOXA cluster for three EZH2 ChIP data sets. Kaneko et al. (2013) used an EZH2 antibody generated in-house; Mu et al. (2018) and Cherney (this study) used EZH2-CS5246. (B) Peak overlap and (C) correlation of signal under Cherney peaks between EZH2 ChIP-Seq data sets. Cherney peak locations are listed in Table S5.

Because EZH2-CS5246 has been used in hundreds of publications (661 at the time of this writing), we wanted to verify that the antibody retrieves PRC2-specific signal by ChIP-Seq. We note that the wash conditions we used for RIP-Seq in this study are either identical or similar to wash conditions previously used for EZH2-CS5246 ChIP-Seq in our own and other works (Deaton and Bird, 2011; Kaneko et al., 2013; Mu et al., 2018; Schertzer et al., 2019a). We performed ChIP-Seq with EZH2-CS5246 in ESCs and compared our data to two previous studies in ESCs, one using EZH2-CS5246 (Mu et al., 2018) and another using an EZH2 antibody generated in-house (Kaneko et al., 2013) (Table S5). The three datasets were highly concordant by eye (Fig. 4A), identified near-complete overlap in peak locations (Fig. 4B), and exhibited highly significant correlations when examining signal under peaks (Fig. 4C), supporting the view that EZH2-CS5246 can be used to generate PRC2-specific signal in ChIP assays.

PRC2 exhibits an enigmatic relationship with RNA (Trotman et al., 2021). By performing IP with a rabbit monoclonal antibody that is highly specific for EZH2 by western blot and yields specific signal for PRC2 by ChIP, we retrieved SAFB, a chromatin-associated RNA-binding protein that has previously been implicated in gene silencing and PRC2 function, and which has been previously shown to associate with *Xist/XIST* (Townson et al., 2004; Mukhopadhyay et al., 2014; Chu et al., 2015; Bousard et al., 2019; Huo et al., 2020; McCarthy et al., 2021; Yu et al., 2021). We find that the RNA enriched in EZH2-CS5246 RIPs is SAFB- and not EZH2-dependent. SAFB or other RNA-binding proteins may yet prove to be important for RNA-mediated recruitment of PRC2 to chromatin. Indeed, RBFOX2 and HNRNPA2B1 may be two such proteins (Meredith et al., 2016; Wei et al., 2016). However, continued work in this area is needed to arrive at mechanistic clarity. Our study highlights the importance of orthogonal assays when interpreting interactions between RNA and chromatin-modifying enzymes, including and not limited to PRC2.

## MATERIALS AND METHODS

### Experimental methods

#### Cell culture

Male mouse ESCs expressing doxycycline-inducible *Xist* from the *Rosa26* locus [derivation described in Trotman et al. (2020)] were grown in DMEM (Gibco) supplemented with 15% Qualified Fetal Bovine Serum (Gibco), 1% Pen/Strep (Gibco), 1% L-Glutamine (Gibco), 1% Non-Essential Amino Acids (Gibco), 100 μM betamercaptoethanol (Sigma-Aldrich) and 0.2% LIF. Cells were passaged every other day and maintained in incubators set at 37°C and 5% CO_2_. Media was replaced daily.

#### Nuclear extraction of ESCs

ESC nuclear extracts were prepared as in Carey et al. (2009). All nuclear extraction steps were performed at 4°C. Briefly, cells were grown to ∼80% confluency in 15 cm plates and washed twice in cold 1xPBS. 10 ml of 1xPBS supplemented with 0.5 mM PMSF (Thermo Fisher Scientific, #36978) per plate were scraped and consolidated to a 15 ml conical tube. Cells were spun at 1000 rpm for 5 min. PBS was aspirated and cells were resuspended in 2× Packed Cell Volume of hypotonic cell lysis buffer (10 mM HEPES-KOH pH 7.9, 1.5 mM MgCl_2_, 10 mM KCl) supplemented with 0.5 mM DTT (Thermo Fisher Scientific, #15508013), 0.5 mM PMSF (Thermo Fisher Scientific, #36978) and 1× Protease Inhibitor Cocktail (PIC; Sigma Product, #P8340); ex: 100 μl of pelleted cells would be resuspended in 200 μl hypotonic cell lysis buffer with supplements. NP-40 was added to the cell resuspension to a final concentration of 0.1% and pellet was incubated for 5 min. Resuspended cell pellet was transferred to a B-Dounce and was lysed via Dounce homogenizer ten times, after which the lysed cells were spun down for 4 min at 2000 rpm and supernatant removed. Cells were then resuspended in 1.5× Packed Cell Volume of nuclear lysis buffer (20 mM HEPES-KOH pH 7.9, 25% Glycerol, 420 mM KCl, 1.5 mM MgCl_2_, 0.2 mM EDTA) supplemented with 0.5 mM DTT (Thermo Fisher Scientific, #15508013), 0.5 mM PMSF (Thermo Fisher Scientific, #36978) and 1× PIC (Sigma Product, #P8340) and incubated for 30 min with rotation, then spun down for 5 min at 5000 rpm. Supernatant was moved to a new tube and extract was fully cleared by spinning at top speed for 15 min. Nuclear extraction was repeated by pellet resuspension in 1.5× Packed Cell Volume of nuclear lysis buffer supplemented with 0.5 mM DTT (Thermo Fisher Scientific, #15508013), 0.5 mM PMSF (Thermo Fisher Scientific, #36978) and 1× PIC (Sigma Product, #P8340) and incubated for 30 min with rotation, then spun down for 5 min at 5000 rpm. Supernatant was moved to a new tube and extract was cleared by spinning at top speed for 15 min. Nuclear extract was then dialyzed for 2 h in Dialysis Buffer (20 mM HEPES-KOH pH 7.9, 20% Glycerol, 100 mM KCl, 0.2 mM EDTA) supplemented fresh 0.2 mM DTT, on a rotating platform at 4°C using a Mini-Dialyzer (Thermo Fisher Scientific, #88403). After 2 h, dialysis buffer with 0.2 mM DTT was replaced, and samples were dialyzed overnight on a rotating platform at 4°C. The next day supernatant was cleared by spinning down at high speed for 15 min. Samples were snap frozen in 200 μl aliquots in liquid N_2_ and stored at −80°C until use.

#### IP-MS sample preparation

40 μl protein A/G agarose beads (Santa Cruz Biotechnology, sc-2003) were washed three times in blocking buffer (0.5% BSA in 1xPBS) and incubated overnight at 4°C with 20 μl EZH2 antibody (anti-EZH2, Cell Signaling Technology, #5246) or 20 μg IgG (anti-IgG; Invitrogen, #02-6102). Beads were then washed three times in fRIP buffer (25 mM Tris-HCl pH 7.5, 5 mM EDTA, 0.5% NP-40, 150 mM KCl). To the beads, 3.2 mg of wild-type (WT) ESC or EZH2 KO ESC nuclear extract was added. RNase treated nuclear extracts were incubated with 1 μl:40 μl ratio of RNase (Thermo Fisher Scientific, #EN0531) to nuclear extract for 1 h at 4°C with rotation before being added to the antibody conjugated beads. Physiological salt levels (175 mM) were recapitulated by bringing nuclear extracts to a total of 2.5 ml in 1:1 RIPA (50 mM Tris-HCl, pH8.0, 1% Triton X-100, 0.5% sodium deoxycholate, 0.1% SDS, 5 mM EDTA, 150 mM KCl) and fRIP buffers (25 mM Tris-HCl pH 7.5, 5 mM EDTA, 0.5% NP-40, 150 mM KCl), supplemented with 1× PIC (PIC; Sigma Product, #P8340), 12.5 μl SuperaseIN (Thermo Fisher Scientific, #AM2696), and 0.5 mM DTT (Thermo Fisher Scientific, #15508013). Samples were rotated overnight at 4°C and then washed once with 1 ml fRIP buffer, resuspended in 1 ml PolII ChIP Buffer (50 mM Tris-HCl pH 7.5, 140 mM NaCl, 1 mM EDTA, 1 mM EGTA, 1% Triton X-100, 0.1% Na-deoxycholate, 0.1% SDS), and transferred to a new tube. Samples were then rotated for 5 min and spun down at 2000 rpm for 1 min. Samples were washed two more times with PolII ChIP Buffer, once with High Salt ChIP Buffer (50 mM Tris-HCl pH7.5, 500 mM NaCl, 1 mM EDTA, 1 mM EGTA, 1% Triton X-100, 0.1% Na-deoxycholate, 0.1% SDS), and resuspended in 1 ml LiCl buffer (20 mM Tris pH 8.0, 1 mM EDTA, 250 mM LiCl, 0.5% NP-40, 0.5% Na-deoxycholate), with a 5 min rotation and 1 min spin down at 2000 rpm after each wash. Samples were moved to a new tube at the final LiCl wash. After aspiration of the LiCl buffer, samples were resuspended in cold 1xPBS and moved to a new tube. Samples were washed three times with 1 ml cold 1xPBS. 25% of samples were saved for western blot, and the remaining 75% were subjected to on-bead trypsin digestion. (Rank et al., 2021). Briefly, after the last wash buffer step during affinity purification, beads were resuspended in 50 μl of 50 mM ammonium bicarbonate (pH8). On-bead digestion was performed by adding 50 μl 50 mM ammonium bicarbonate (pH8) and 1 μg trypsin and incubated, shaking, overnight at 37°C. Beads were pelleted and transferred supernatants to fresh tubes. The beads were washed twice with 100 μl LC-MS grade water, and washes added to the original supernatants. Samples were acidified by adding formic acid to final concentration of 2%, to pH ∼2. Peptides were desalted using peptide desalting spin columns (Thermo Fisher Scientific), lyophilized, and stored at −80°C until further analysis.

#### LC/MS/MS analysis

The peptide samples were analyzed by LC/MS/MS using an Easy nLC 1200 coupled to a QExactive HF Biopharma mass spectrometer (Thermo Fisher Scientific). Samples were injected onto an Easy Spray PepMap C18 column (75 μm id×25 cm, 2 μm particle size) (Thermo Fisher Scientific) and separated over a 2 h method. The gradient for separation consisted of 5-45% mobile phase B at a 250 nl/min flow rate, where mobile phase A was 0.1% formic acid in water and mobile phase B consisted of 0.1% formic acid in acetonitrile (ACN). The QExactive HF was operated in data-dependent mode where the 15 most intense precursors were selected for subsequent fragmentation. Resolution for the precursor scan (m/z 350-1700) was set to 60,000, while MS/MS scans resolution was set to 15,000. The normalized collision energy was set to 27% for HCD. Peptide match was set to preferred, and precursors with unknown charge or a charge state of 1 and ≥7 were excluded.

#### Immunoprecipitation followed by western blot (IP-western)

25 μl protein A/G agarose beads (Santa Cruz Biotechnology, sc-2003) were washed three times in blocking buffer (0.5% BSA in 1xPBS) and incubated overnight at 4°C with 7.5 μl antibody (anti-EZH2, Cell Signaling Technology, #5246; SAFB, Bethyl A300-812A; SUZ12, Cell Signaling Technology, #3737) or 3 μl (7.5 μg) IgG (anti-IgG; Invitrogen, #02-6502). The following day, beads were washed twice in blocking buffer and resuspended in 300 μg nuclear extract. Nuclear extract was diluted with a 1:1 mixture of RIPA (50 mM Tris-HCl, pH8.0, 1% Triton X-100, 0.5% sodium deoxycholate, 0.1% SDS, 5 mM EDTA, 150 mM KCl) and fRIP buffers (25 mM Tris-HCl pH 7.5, 5 mM EDTA, 0.5% NP-40, 150 mM KCl) to bring salt concentrations to physiological levels (175 mM). Nuclear extract mixture was supplemented with 1 mM PMSF (Thermo Fisher Scientific, #36978) and 1× Protease Inhibitor Cocktail (PIC; Sigma Product, #P8340) and samples rotated overnight at 4°C. The next day, samples were washed four times in a 1:1 mixture of RIPA (50 mM Tris-HCl, pH8.0, 1% Triton X-100, 0.5% sodium deoxycholate, 0.1% SDS, 5 mM EDTA, 150 mM KCl) and fRIP buffers (25 mM Tris-HCl pH 7.5, 5 mM EDTA, 0.5% NP-40, 150 mM KCl) to bring salt concentrations to physiological levels (175 mM). Nuclear extract mixture was supplemented with 1 mM PMSF (Thermo Fisher Scientific, #36978) and 1× Protease Inhibitor Cocktail (PIC; Sigma Product, #P8340). Tubes were changed for the first and the last wash. After the last wash, beads were spun down and resuspended in 75 μl 4xSDS loading buffer [Sigma-Aldrich Recipe: 0.2 M Tris-HCl pH6.8, 0.4 M DTT, 8% (w/v) SDS, 6 mM Bromophenol Blue, 4.3 M Glycerol] diluted to 1×, and 20 μl loaded for western blotting.

#### Whole-cell western blots

To isolate protein for western blotting, 0.8×10e6 cells were washed with 1xPBS and then lysed with 500 μl RIPA buffer (10 mM Tris-Cl pH7.5, 1 mM EDTA, 0.5 mM EGTA, 1% NP40, 0.1% sodium deoxycholate, 0.1% Sodium Dodecyl Sulfate, 140 mM Sodium Chloride) supplemented with 1 mM PMSF (Thermo Fisher Scientific, #36978) and 1×PIC (Sigma Product #P8340). Cell suspensions were rotated for 15 min at 4°C, then spun down at high speed at 4°C for 15 min and supernatant was collected. Prior to western blotting, protein levels were quantified using the DC assay from Bio-Rad (Product #5000006). 4×SDS loading buffer [Sigma-Aldrich Recipe: 0.2 M Tris-HCl pH6.8, 0.4 M DTT, 8% (w/v) SDS, 6 mM Bromophenol Blue, 4.3 M Glycerol] was added to samples to 1× final concentration. Samples were then boiled for 5 min at 95°C, and equal μg (whole cell lysate) or μL (immunoprecipitation) amounts were loaded onto Bio-Rad TGX Stain Free Gels. Samples were run at 50 V until past stacking gel, then at 150 V for 1-2 h. Gels were transferred to PVDF (Immobulon, #IPVH00010) membrane either for 1 h at 125 V at 4°C or overnight at 25 V at 4°C. Membranes were blocked for 45 min in 1xTBST+5% milk (Bio-Rad, #1706404). Membranes were then incubated with primary antibody either overnight at 4°C or for 1-3 h at room temperature (RT). Membranes were washed three times for 5 min each in 1×TBST. Secondary antibodies were diluted in 1xTBST+5% milk and incubated with membranes for 45 min (1:100,000; Invitrogen). Membranes were then washed three times in 1xTBST washes for 10 min, before being imaged with ECL (Thermo Fisher Scientific, #34096). Antibodies used were: EZH2 (Cell Signaling Technology, #5246, 1:1000), EZH2 (Cell Signaling Technology, #3147, 1:1000), SAFB (Bethyl A300-812A, 1:3000), TBP (Abcam ab818, 1:2000), goat anti-mouse (Thermo Fisher Scientific, A16072, 1:100,000) and goat anti-rabbit (Thermo Fisher Scientific, G21234, 1:100,000).

#### Inducible knockdown of SAFB in ESCs

Guide RNAs targeting SAFB were designed using Benchling. sgRNA sequences are found in Table S3. Guides were cloned into the rtTA-BsmbI piggyBac vector from (Schertzer et al., 2019b; Addgene plasmid, #126028). For stable plasmid integration, parent ESCs were seeded at 0.5×10^6^ cells per well in a six-well plate. The following day, the cells were transfected using Lipofectamine 3000 (Invitrogen, #L3000-015), using the following amounts of reagent: 1818 ng of sgRNA or empty plasmid, 455 ng Cas9 plasmid, and 228 ng Transposase (2.5 μg total), mixed with 5 μl P3000 reagent, 7.5 μl Lipofectamine 3000 reagent, and with Opti-MEM media (Gibco, #31985-070) to final volume of 250 μl. The reagents were incubated for 5 min at RT before being added to cells with fresh media. After 24 h, cells were co-selected with Hygromycin (150 μg/ml) for 1 week and G418 (200 μg/ml) for 12 days. To deplete SAFB from cells, fresh doxycycline was added to media daily at 1 μg/ml for 3 days. Media was replaced daily.

#### Generation of EZH2 knockout ESCs

sgRNAs to delete the EZH2 locus were designed to the mm10 genome using CRISPOR with the specifications: 20bp-NGG – Sp Cas9, Sp Cas9-Hf1, eSp Cas9 1.1 (Concordet and Haeussler, 2018). sgRNA sequences are found in Table S3. Guides were cloned into the pX330 plasmid as specified in (Cong et al., 2013; Addgene plasmid #42230). To delete EZH2, Parent ESCs were seeded at 0.5×10^6^ cells per well in a six-well plate. The following day, the cells were transiently transfected using Lipofectamine 3000 (Invitrogen, #L3000-015): 800 ng of sgRNA plasmid pool, or as a control, empty pX330 plasmid, and 200 ng puro resistant GFP plasmid (1 μg total) were mixed with 2 μl P3000 reagent, 7.5 μl Lipofectamine 3000 reagent and with Opti-MEM media (Gibco, #31985-070) to final volume of 250 μl. The reagents were incubated for 5 min at RT before being added to cells with fresh media. After 24 h, cells were pulsed with Puromycin (2 μg/ml) for 48 h. After puromycin selection, cells were trypsinized to single cells and plated onto irradiated fibroblast feeder cells (500-2000 cells/10 cm plate) until individual colonies were visible by eye (4-5 days). Individual colonies were then selected and grown in individual wells for genotyping.

The two KO lines that were selected for further study, along with the WT control line, were then rendered dox-inducible by transfection of the rtTA-expressing plasmid described in Kirk et al. (2018). One day prior to transfection, parent and KO ESCs were seeded at 0.5×10^6^ cells per six-welled well. The following day, 500 ng of rtTA plasmid and 500 ng of transposase (1 μg total DNA) were mixed with 2 μl P3000 reagent, 7.5 μl Lipofectamine 3000 reagent and with Opti-MEM media (Gibco, #31985-070) to 250 μl. The reagents incubated for 5 min at RT before being added to cells with fresh media. After 24 h, cells underwent G418 selection (200 μg/ml) for 12 days. Guides have been deposited to Addgene.

#### PCR

Genomic DNA was collected from 0.8×10^6^ cells with 500 μl lysis buffer (100 mM Tris-HCl pH8.1, 0.5 mM EDTA pH 8.0, 200 nM NaCl, 0.2% SDS)+80 μl Proteinase K (Denville)+8 μl linear acrylamide (Thermo Fisher Scientific, #AM9520) and incubated at 55°C overnight. Twice the volume of ice cold 100% ethanol was added. Samples were then vortexed and rotated at 4°C for 15 min. Samples were spun at max speed for 5 min at 4°C. The lysis buffer/ethanol mixture was then removed, and the DNA pellet was washed with 70% ethanol, after which the DNA pellet was resuspended in 1×TE (10 mM Tris pH8.0, 1 mM EDTA) and incubated overnight at 56°C. DNA concentration was measured via Nanodrop and diluted to 50 ng/μl. PCR was performed with ChoiceTAQ (Denville CB4050) as follows: 25 μl PCR reaction mixture (2.5 μl 10× PCR reaction buffer, 0.2 μl 10 mM dNTPs, 0.25 μl 100uM primers, 0.25 μl Choice TAQ polymerase, 3 μl DNA template (50 ng/μl) and ddH2O to 25 μl) ran in Bio-Rad C1000 Touch or T100 thermocycler (initial denaturation at 95°C for 3 min; 25 cycles of 95°C for 30 s, 60-63°C annealing for 30 s, and 72°C for 30-45 s extension time). PCR primers and conditions are in Table S3.

#### Total RNA isolation

ESCs were grown in six-well plates to ∼80% confluency. Cells were washed twice with 1×PBS and 1 ml of TRIzol (Thermo Fisher Scientific, #15596018) was added per well. Samples were pipetted up and down at least ten times, transferred to a microcentrifuge tube and briefly vortexed. Samples were incubated at RT for 5 min then 200 μl of chloroform was added. Afterwards, samples were vortexed and incubated for 3 min at RT. Samples were spun down at 12,000 rpm for 15 min at 4°C. The upper aqueous phase was moved to a new tube and 8 μl of linear acrylamide (Thermo Fisher Scientific, #AM9520) was added. Then 500 μl of 100% isopropanol was added, and samples were vortexed and incubated at RT for 10 min. Tubes were spun down at 12,000 rpm for 10 min at 4°C. Supernatant was removed and pellets washed with 1 ml of cold 75% ethanol. Samples were briefly vortexed and spun down at 7500 rpm for 5 min at 4°C. Supernatant was discarded and pellet was dried by repeated spin down and aspiration. Final pellets were resuspended in 100 μl water.

#### RT-qPCR

Equal amounts of RNA (0.5-1μg) were reverse transcribed using the High-Capacity cDNA Reverse Transcription Kit (Thermo Fisher Scientific, #4368813) with the random primers provided, and then diluted with 30μL 1xTE. For RIP RT-qPCR, 2 μl of eluted sample were used in RT reactions. 10 μl qPCR reactions were performed using iTaq Universal SYBR Green (Bio-Rad) and custom primers on a Bio-Rad CFX96 system with the following thermocycling parameters: initial denaturation at 95°C for 10 min; 40 cycles of 95°C for 15 s, 60°C for 30 s, and 72°C for 30 s followed by a plate read. The primer concentration used for all qPCR reactions in this study was 0.5μM. Standard curves were used in all qPCR analyses and were prepared by RT of equal volume of WT sample to other samples. After RT, five 5-fold serial dilutions were made (six total standards including undiluted RT reaction) and added in duplicate to qPCR plates. After qPCR run, samples were normalized to standard curve read using the Bio-Rad CFX Manager Software. See Table S3 for all primer sequences used.

#### Antibodies

All antibodies and their conditions used for this study are listed in Table S4.

#### Formaldehyde crosslinking of ESCs

For RIP, cells were grown to 75-85% confluency, trypsinized and counted. Cells were washed twice in cold 1xPBS then rotated for 30 min in 10 ml of 0.3% formaldehyde [1 ml 16% methanol-free formaldehyde (Pierce, #28906) in 49 ml 1xPBS] at 4°C. Formaldehyde was quenched with 1 ml of 2 M glycine for 5 min at RT with rotation. Cells were washed three times in cold 1xPBS, then resuspended in 1xPBS at 10×10^6^ cells per ml, aliquoted 10×10^6^ cells to new tubes, and spun down. PBS was aspirated and pellets were snap frozen in a liquid nitrogen bath and immediately transferred to −80°C until use.

#### Formaldehyde+DSG crosslinking of ESCs for EZH2-CS5246 ChIP

Cells were grown to 80% confluency and washed three times in RT 1xPBS. Cells were scraped and transferred to conical tubes, spun down and supernatant removed. Cells were first crosslinked with 15 ml of 2 mM DSG (Thermo Fisher Scientific, #20593) in 1xPBS and rotated at RT for 45 min. Cells were then washed two times in RT 1xPBS and then crosslinked for 15 min at room temperature with 15 ml of 1% formaldehyde (Thermo Fisher Scientific, #28906) with rotation. Formaldehyde was quenched with 1.67 ml of 2.5 M glycine for 5 min with rotation. Conical tubes were placed on ice and cells were washed twice with cold 1xPBS and then spun down at 3000 rpm at 4°C. Cell pellets were resuspended in 1xPBS supplemented with 1 mM PMSF and 1×PIC to a concentration of 10×10^6^ cells/ ml. Cells were aliquoted 10×10^6^ cells to new tubes. PBS was spun out at 1200 rpm for 5 min at 4°C. PBS was aspirated, and cells were flash frozen with a dry ice-methanol bath and stored at −80°C until use.

#### RIPs

RIPs were performed similar to Schertzer et al. (2019a), which is a protocol originally adapted from Hendrickson et al. (2016); Raab et al. (2019). 25 μl protein A/G agarose beads (Santa Cruz Biotechnology, #sc-2003) were washed three times in blocking buffer (0.5% BSA in 1xPBS) and incubated overnight at 4°C with 10 μl antibody (anti-SAFB, Bethyl 812-300A; EZH2, Cell Signaling Technology, #5246) or 10μg IgG (Invitrogen, #02-6502). 10×10^6^ cells were resuspended in 500 μl RIPA Buffer (50 mM Tris-HCl, pH8, 1% Triton X-100, 0.5% sodium deoxycholate, 0.1% SDS, 5 mM EDTA, 150 mM KCl) supplemented with 1×PIC (Sigma-Aldrich, #P8340), 2.5 μl SuperaseIN (Thermo Fisher Scientific, #AM2696) and 0.5 mM DTT (Thermo Fisher Scientific, #15508013) and sonicated twice for 30 s on and 1 min off at 30% output using the Sonics Vibracell Sonicator (Model VCX130, Serial# 52223R). Samples were spun down at high speed for 15 min and 50 μl total lysate was saved for input. Beads were washed three times in 1 ml fRIP buffer (25 mM Tris-HCl pH7.5, 5 mM EDTA, 0.5% NP-40, 150 mM KCl) and resuspended in 450 μl fRIP buffer supplemented as above with PIC, SuperaseIN and DTT, then mixed with sonicated samples. Samples were rotated overnight at 4°C, then washed once with 1 ml fRIP buffer and resuspended in 1 ml PolII ChIP Buffer (50 mM Tris-HCl pH 7.5, 140 mM NaCl, 1 mM EDTA, 1 mM EGTA, 1% Triton X-100, 0.1% Sodium-deoxycholate, 0.1% Sodium dodecyl sulfide) before being transferred to a new tube. Samples were rotated at 4°C for 5 min, spun down at 1200 rpm, and the supernatant aspirated. Samples were washed twice more with 1 ml PolII ChIP Buffer, once with 1 ml High Salt ChIP Buffer (50 mM Tris-HCl pH7.5, 500 mM NaCl, 1 mM EDTA, 1 mM EGTA, 0.1% sodium-deoxycholate, 0.1% sodium dodecyl sulfide, 1% Triton X-100), and once in 1 ml LiCl buffer (20 mM Tris pH8.0, 1 mM EDTA, 250 mM LiCl, 0.5% NP-40, 0.5% sodium-deoxycholate); each wash included a 5-min rotation at 4°C. At the final wash, samples were transferred to a new tube. After the final wash, inputs were thawed on ice and bead samples were resuspended in 56 μl water, 33 μl of three times reverse-crosslinking buffer (3×PBS, 6% N-lauroyl sarcosine and 30 mM EDTA), 5 μl 100 mM DTT (Thermo Fisher Scientific, #15508013), 20 μl Proteinase K (Thermo Fisher Scientific, #25530015), and 1 μl of SuperaseIN. Samples were incubated for 1 h at 42°C, 1 h at 55°C, 30 min at 65°C, and mixed by pipetting every 15 min. Afterwards, 1 ml Trizol (Thermo Fisher Scientific, #15596018) was added, samples were vortexed, 200 μl chloroform was added, samples were vortexed again, and finally spun at 12,000 rpm for 15 min at 4°C. The aqueous phase was then extracted and to that one volume of 100% ethanol was added. Samples were vortexed and applied to Zymo-Spin IC Columns (Zymo, #R1014) and spun for 30 s at top speed on a benchtop microcentrifuge. 400 μl of RNA Wash Buffer (Zymo, #R1014) was added and samples were spun at top speed for 30 s. For each sample, 5 μl DNase I (Zymo, #R1014) and 35 μl of DNA Digestion Buffer (Zymo, #R1014) was added directly to the column matrix and incubated at room temp for 20 min. 400 μl of RNA Prep Buffer (Zymo, #R1014) was then added to the columns, and columns were spun at top speed for 30 s. 700 μl RNA Wash Buffer (Zymo, #R1014) was then added, and columns were spun at top speed for 30 s. 400 μl RNA Wash Buffer was then added, and columns were spun at top speed for 30 s. The flow through was discarded and columns spun again for 2 min to remove all traces of wash buffer. Columns were transferred to a clean tube, 15 μl of ddH2O was added to each column, and after a 5-min incubation, samples were spun at top speed for 2 min to elute.

#### RNA sequencing

For RIP-Seq samples, 9 μl of RIP sample (from 15 μl total) were used. Each library preparation included 1 μl of 1:250 dilution of ERCC Spike-In RNAs (Ambion, #4456653). 10 μl total were prepped using the KAPA RNA HyperPrep Kit with RiboErase (Kapa Biosystems; product #KR1351). Sequencing was performed on an Illumina Next-Seq 500, using high-output, 75-cycle kits (Illumina, #20024906).

#### EZH2 ChIP-Seq

The day before sonication, 25 μl of protein A/G agarose beads (Santa Cruz Biotechnology, #sc-2003) were washed three times in block solution (0.5% BSA in 1xPBS) before being resuspended in 300 μl blocking solution. 10 μl EZH2 antibody (Cell Signaling Technology, #5246) per 10 million cells was added, then beads and antibody conjugated via rotation overnight at 4°C. On the day of sonication, 10 million ESCs crosslinked with DSG and 1% formaldehyde were resuspended in PolII ChIP Buffer (no Triton) (50 mM Tris-HCl, pH 7.5, 140 mM NaCl, 1 mM EDTA, pH 8.0, 1 mM EGTA, pH 8., 0.1% Sodium-deoxycholate, 0.1% Sodium dodecyl sulfide) supplemented with 1×PIC (Sigma Product, #P8340) and then sonicated ten times for 30 s on and 1 min off at 30% output using the Sonics Vibracell Sonicator (Model VCX130, Serial# 52223R). Supernatant was cleared by top speed centrifugation for 20 min, moved to a new tube and 10% Triton-X100 added to a final 1%. 10% of final volume was taken and saved as input. Conjugated antibody/bead mixture was spun down and washed three times in blocking buffer. ChIPs were then performed by incubating sonicated cell lysates with pre-conjugated EZH2 antibody/agarose beads overnight at 4°C. The next day, samples were spun down and resuspended 1 ml PolII ChIP Buffer (50 mM Tris-HCl pH 7.5, 140 mM NaCl, 1 mM EDTA, 1 mM EGTA, 1% Triton X-100, 0.1% Sodium-deoxycholate, 0.1% Sodium dodecyl sulfide) before being transferred to a new tube. Samples were rotated at 4°C for 5 min, spun down at 2000 rpm for 2 min, and the supernatant aspirated. Samples were washed twice more with 1 ml PolII ChIP Buffer, once with 1 ml High Salt ChIP Buffer (50 mM Tris-HCl pH 7.5, 500 mM NaCl, 1 mM EDTA, 1 mM EGTA, 0.1% sodium-deoxycholate, 0.1% sodium dodecyl sulfide, 1% Triton X-100), once in 1 ml LiCl buffer (20 mM Tris pH 8.0, 1 mM EDTA, 250 mM LiCl, 0.5% NP-40, 0.5% sodium-deoxycholate), and once in 1xTE; each wash included a 5-min rotation at 4°C. At the final wash, samples were transferred to a new tube. To elute the DNA, beads were re-suspended in 250 μl Elution buffer (50 mM Tris pH 8.0, 10 mM EDTA, and 1% SDS) and placed on a 65°C heat block for 17 min with vortexing every 2-3 min. Supernatant was moved to a new tube, 240 μl and 10 μl 5 M NaCl, and crosslinks were reversed overnight at 65°C. The following day, eluates were incubated with 3 μl RNase A (Thermo Fisher Scientific, EN0531) for 1 h at 37°C and then 10 μl Proteinase K (Thermo Fisher Scientific, #25530015) for 2-3 h at 56°C. One volume (510 μl) of phenol:chloroform:isoamyl alcohol (Sigma-Aldrich, P3803) was added to the DNA, and was spun at max speed for 5 min at 4°C. The aqueous solution was transferred to a new tube. 5 μl of linear acrylamide was added, and then 1/10 volume (50 μl) of 3 M NaOAc, pH5.4. Samples were vortexed and spun down. 2 volumes (1 ml) of ice cold 100% ethanol were added, and samples were vortexed, spun down and incubated overnight at −20°C. The next day, samples were spun at max speed for 30 min at 4°C (cold room). Supernatant was aspirated and DNA pellet was washed in 1 ml ice-cold 80% ethanol. Samples were spun down and supernatant was removed. Samples were dried by repeated aspiration and pulse spin. Samples were resuspended in 30 μl 1X TE for sequencing. DNA was prepared for sequencing on the Illumina platform using Next Reagents (NEB) and Agencourt AMPure XP beads (Beckman Coulter).

### Computational analyses

#### Mass spectrometry data analysis

Raw data files were processed using MaxQuant version 1.6.12.0 and searched against the reviewed mouse database (containing 17,051 entries), appended with a contaminants database, using Andromeda within MaxQuant. Enzyme specificity was set to trypsin, up to two missed cleavage sites were allowed, and methionine oxidation and N-terminus acetylation were set as variable modifications. A 1% FDR was used to filter all data. Match between runs was enabled (5 min match time window, 20 min alignment window), and a minimum of two unique peptides was required for label-free quantitation using the LFQ intensities. Perseus was used for further processing (Tyanova et al., 2016). Only proteins with >1 unique+razor peptide were used for LFQ analysis. Proteins with 50% missing values were removed and missing values were imputed from normal distribution within Perseus. Log2 fold change (FC) ratios were calculated using the averaged Log2 LFQ intensities of IP sample compared to IgG control, and Student’s *t*-test performed for each pairwise comparison, with *P*-values calculated. Proteins with significant *P*-values (<0.05) and Log2 FC >1 were analyzed further. All analyzed protein interaction data are present in Table S1. The mass spectrometry proteomics data have been deposited to the ProteomeXchange Consortium via the PRIDE partner repository (Perez-Riverol et al., 2022) with the dataset identifier PXD038103.

#### RIP-Seq alignment

RIP-Seq data were aligned to the mm10 mouse genome using STAR with default parameters (Dobin et al., 2013). Alignments with a quality score ≥30 were retained for subsequent analyses (Li et al., 2009).

#### RIP-Seq peak calling

SAFB peaks were identified as part of (Cherney et al., 2022 preprint). For EZH2 RIP-Seq peak calling, after alignment and filtering, all EZH2 RIP-Seq data from WT ESC replicates were concatenated and using samtools, split into two files, corresponding to alignments that mapped to the positive and negative strands of the genome, respectively. Using a custom perl script, the strand information within the positive and negative strand alignment files was randomized so as to better match the criteria of the MACS peak caller, which uses the average distance between positive and negative strand alignments to estimate the fragment length (Zhang et al., 2008). Putative peaks were called on strand-randomized positive and negative strand alignment files, respectively, using default MACS parameters and not providing a background file (Zhang et al., 2008). Peak bed files were converted to SAF format and reads under each putative peak were counted from EZH2 RIP-Seq alignments performed in WT, EZH2 KO, and SAFB/2 DKO ESCs using featureCounts (Liao et al., 2014). EZH2 peaks were determined by retaining putative peaks that were represented by at least five reads in at least two of the three WT ESC lines profiled. We retained only those putative peaks that harbored an average aligned-reads-per-million-total-reads (RPM) signal of at least 5-fold less in IgG RIPs compared to WT EZH2 RIPs. This yielded 33,671 regions that were enriched in their association with EZH2 in WT ESCs. (Table S2).

#### Significance of RIP-Seq peak overlaps

To determine the significance of RIP-Seq peak overlaps, the hypergeometric test in R (2023) was used under the following conditions: phyper(q=overlap, m=set1 (post-filter), *n*=total peaks (pre-filter) – set1 (post-filter), k=set2 (post-filter), lower.tail=FALSE).

#### RIP-Seq scatter plots

Scatter plots in Fig. 2 were constructed using featureCounts to count the reads under each of the 30,998 SAFB peaks in each dataset (Liao et al., 2014). Read counts were then plotted using R (2023).

#### UCSC wiggle density plots

UCSC wiggle density plots were made from filtered sam files using custom perl scripts normalized to reads per million (rpm). Tracks of pooled replicates are located here: https://genome.ucsc.edu/s/recherney/Cherney_Antibody_2023.

#### ChIP-Seq peak calling

EZH2 data were aligned to mm10 using bowtie2 (Langmead and Salzberg, 2012). Peaks were called using MACS2 under the following parameters using an H3 control: [macs2 callpeak -t -c bam -n -f BAM -g mm –broad –broad-cutoff 0.01] (Zhang et al., 2008). EZH2 peak locations are included in Table S5.

#### ChIP-Seq scatter plots

Scatter plots in Fig. 4 were constructed using featureCounts to count the reads from each EZH2 ChIP-seq data set under each of the EZH2 Cherney peaks (Table S5; Liao et al., 2014). Read counts were then plotted using R (2023).

#### Sequencing data

Sequencing data are available under the GEO accession number GSE227893.

## Supplementary Material

10.1242/biolopen.059955_sup1 Supplementary information

## References

[BIO059955C1] Beltran, M., Yates, C. M., Skalska, L., Dawson, M., Reis, F. P., Viiri, K., Fisher, C. L., Sibley, C. R., Foster, B. M., Bartke, T. et al. (2016). The interaction of PRC2 with RNA or chromatin is mutually antagonistic. *Genome Res.* 26, 896-907. 10.1101/gr.197632.115 27197219 PMC4937559

[BIO059955C2] Beltran, M., Tavares, M., Justin, N., Khandelwal, G., Ambrose, J., Foster, B. M., Worlock, K. B., Tvardovskiy, A., Kunzelmann, S., Herrero, J. et al. (2019). G-tract RNA removes Polycomb repressive complex 2 from genes. *Nat. Struct. Mol. Biol.* 26, 899-909. 10.1038/s41594-019-0293-z 31548724 PMC6778522

[BIO059955C3] Bousard, A., Raposo, A. C., Zylicz, J. J., Picard, C., Pires, V. B., Qi, Y., Gil, C., Syx, L., Chang, H. Y., Heard, E. et al. (2019). The role of Xist-mediated Polycomb recruitment in the initiation of X-chromosome inactivation. *EMBO Rep.* 20, e48019. 10.15252/embr.201948019 31456285 PMC6776897

[BIO059955C4] Cao, Q., Wang, X., Zhao, M., Yang, R., Malik, R., Qiao, Y., Poliakov, A., Yocum, A. K., Li, Y., Chen, W. et al. (2014). The central role of EED in the orchestration of polycomb group complexes. *Nat. Commun.* 5, 3127. 10.1038/ncomms4127 24457600 PMC4073494

[BIO059955C5] Carey, M. F., Peterson, C. L. and Smale, S. T. (2009). Dignam and Roeder nuclear extract preparation. *Cold Spring Harb. Protoc.* 2009, pdb.prot5330. 10.1101/pdb.prot5330 20150077

[BIO059955C6] Cherney, R. E., Eberhard, Q. E., Mills, C. A., Porrello, A., Zhang, Z., White, D., Herring, L. E. and Calabrese, J. M. (2022). SAFB associates with nascent RNAs to promote gene expression in mouse embryonic stem cells. *bioRxiv*. 10.1261/rna.079569.122 PMC10578485 37468167

[BIO059955C7] Chu, C., Zhang, Q. C., Da Rocha, S. T., Flynn, R. A., Bharadwaj, M., Calabrese, J. M., Magnuson, T., Heard, E. and Chang, H. Y. (2015). Systematic discovery of Xist RNA binding proteins. *Cell* 161, 404-416. 10.1016/j.cell.2015.03.025 25843628 PMC4425988

[BIO059955C8] Cifuentes-Rojas, C., Hernandez, A. J., Sarma, K. and Lee, J. T. (2014). Regulatory interactions between RNA and polycomb repressive complex 2. *Mol. Cell* 55, 171-185. 10.1016/j.molcel.2014.05.009 24882207 PMC4107928

[BIO059955C9] Concordet, J. P. and Haeussler, M. (2018). CRISPOR: intuitive guide selection for CRISPR/Cas9 genome editing experiments and screens. *Nucleic Acids Res.* 46, W242-W245. 10.1093/nar/gky354 29762716 PMC6030908

[BIO059955C10] Cong, L., Ran, F. A., Cox, D., Lin, S., Barretto, R., Habib, N., Hsu, P. D., Wu, X., Jiang, W., Marraffini, L. A. et al. (2013). Multiplex genome engineering using CRISPR/Cas systems. *Science* 339, 819-823. 10.1126/science.1231143 23287718 PMC3795411

[BIO059955C11] Davidovich, C., Zheng, L., Goodrich, K. J. and Cech, T. R. (2013). Promiscuous RNA binding by Polycomb repressive complex 2. *Nat. Struct. Mol. Biol.* 20, 1250-1257. 10.1038/nsmb.2679 24077223 PMC3823624

[BIO059955C12] Davidovich, C., Wang, X., Cifuentes-Rojas, C., Goodrich, K. J., Gooding, A. R., Lee, J. T. and Cech, T. R. (2015). Toward a consensus on the binding specificity and promiscuity of PRC2 for RNA. *Mol. Cell* 57, 552-558. 10.1016/j.molcel.2014.12.017 25601759 PMC4320675

[BIO059955C13] Deaton, A. M. and Bird, A. (2011). CpG islands and the regulation of transcription. *Genes Dev.* 25, 1010-1022. 10.1101/gad.2037511 21576262 PMC3093116

[BIO059955C14] Deevy, O. and Bracken, A. P. (2019). PRC2 functions in development and congenital disorders. *Development* 146, dev181354. 10.1242/dev.181354 31575610 PMC6803372

[BIO059955C15] Dobin, A., Davis, C. A., Schlesinger, F., Drenkow, J., Zaleski, C., Jha, S., Batut, P., Chaisson, M. and Gingeras, T. R. (2013). STAR: ultrafast universal RNA-seq aligner. *Bioinformatics* 29: 15-21. 10.1093/bioinformatics/bts635 23104886 PMC3530905

[BIO059955C16] Gao, Z., Zhang, J., Bonasio, R., Strino, F., Sawai, A., Parisi, F., Kluger, Y. and Reinberg, D. (2012). PCGF homologs, CBX proteins, and RYBP define functionally distinct PRC1 family complexes. *Mol. Cell* 45, 344-356. 10.1016/j.molcel.2012.01.002 22325352 PMC3293217

[BIO059955C17] Garland, W., Comet, I., Wu, M., Radzisheuskaya, A., Rib, L., Vitting-Seerup, K., Lloret-Llinares, M., Sandelin, A., Helin, K. and Jensen, T. H. (2019). A functional link between nuclear RNA decay and transcriptional control mediated by the polycomb repressive complex 2. *Cell Rep.* 29, 1800-1811.e6. 10.1016/j.celrep.2019.10.011 31722198 PMC6856724

[BIO059955C18] Hendrickson, D. G., Kelley, D. R., Tenen, D., Bernstein, B. and Rinn, J. L. (2016). Widespread RNA binding by chromatin-associated proteins. *Genome Biol.* 17, 28. 10.1186/s13059-016-0878-3 26883116 PMC4756407

[BIO059955C19] Hoffman, E. A., Frey, B. L., Smith, L. M. and Auble, D. T. (2015). Formaldehyde crosslinking: a tool for the study of chromatin complexes. *J. Biol. Chem.* 290, 26404-26411. 10.1074/jbc.R115.651679 26354429 PMC4646298

[BIO059955C20] Huo, X., Ji, L., Zhang, Y., Lv, P., Cao, X., Wang, Q., Yan, Z., Dong, S., Du, D., Zhang, F. et al. (2020). The nuclear matrix protein SAFB cooperates with major satellite RNAs to stabilize heterochromatin architecture partially through phase separation. *Mol. Cell* 77, 368-383.e7. 10.1016/j.molcel.2019.10.001 31677973

[BIO059955C21] Kaneko, S., Son, J., Shen, S. S., Reinberg, D. and Bonasio, R. (2013). PRC2 binds active promoters and contacts nascent RNAs in embryonic stem cells. *Nat. Struct. Mol. Biol.* 20, 1258-1264. 10.1038/nsmb.2700 24141703 PMC3839660

[BIO059955C22] Kaneko, S., Son, J., Bonasio, R., Shen, S. S. and Reinberg, D. (2014). Nascent RNA interaction keeps PRC2 activity poised and in check. *Genes Dev.* 28, 1983-1988. 10.1101/gad.247940.114 25170018 PMC4173153

[BIO059955C23] Kirk, J. M., Kim, S. O., Inoue, K., Smola, M. J., Lee, D. M., Schertzer, M. D., Wooten, J. S., Baker, A. R., Sprague, D., Collins, D. W. et al. (2018). Functional classification of long non-coding RNAs by k-mer content. *Nat. Genet.* 50, 1474-1482. 10.1038/s41588-018-0207-8 30224646 PMC6262761

[BIO059955C24] Langmead, B. and Salzberg, S. L. (2012). Fast gapped-read alignment with Bowtie 2. *Nat. Methods* 9, 357-359. 10.1038/nmeth.1923 22388286 PMC3322381

[BIO059955C25] Laugesen, A., Hojfeldt, J. W. and Helin, K. (2016). Role of the polycomb repressive complex 2 (PRC2) in transcriptional regulation and cancer. *Cold Spring Harb. Perspect. Med.* 6, a026575. 10.1101/cshperspect.a026575 27449971 PMC5008062

[BIO059955C26] Li, H., Handsaker, B., Wysoker, A., Fennell, T., Ruan, J., Homer, N., Marth, G., Abecasis, G., Durbin, R. and Genome Project Data Processing, S. (2009). The sequence alignment/map format and SAMtools. *Bioinformatics* 25, 2078-2079. 10.1093/bioinformatics/btp352 19505943 PMC2723002

[BIO059955C27] Liao, Y., Smyth, G. K. and Shi, W. (2014). featureCounts: an efficient general purpose program for assigning sequence reads to genomic features. *Bioinformatics* 30, 923-930. 10.1093/bioinformatics/btt656 24227677

[BIO059955C28] Long, Y., Hwang, T., Gooding, A. R., Goodrich, K. J., Rinn, J. L. and Cech, T. R. (2020). RNA is essential for PRC2 chromatin occupancy and function in human pluripotent stem cells. *Nat. Genet.* 52, 931-938. 10.1038/s41588-020-0662-x 32632336 PMC10353856

[BIO059955C29] Mccarthy, R. L., Kaeding, K. E., Keller, S. H., Zhong, Y., Xu, L., Hsieh, A., Hou, Y., Donahue, G., Becker, J. S., Alberto, O. et al. (2021). Diverse heterochromatin-associated proteins repress distinct classes of genes and repetitive elements. *Nat. Cell Biol.* 23, 905-914. 10.1038/s41556-021-00725-7 34354237 PMC9248069

[BIO059955C30] Meredith, E. K., Balas, M. M., Sindy, K., Haislop, K. and Johnson, A. M. (2016). An RNA matchmaker protein regulates the activity of the long noncoding RNA HOTAIR. *RNA* 22, 995-1010. 10.1261/rna.055830.115 27146324 PMC4911922

[BIO059955C31] Mu, W., Starmer, J., Yee, D. and Magnuson, T. (2018). EZH2 variants differentially regulate polycomb repressive complex 2 in histone methylation and cell differentiation. *Epigenetics Chromatin* 11, 71. 10.1186/s13072-018-0242-9 30522506 PMC6282306

[BIO059955C32] Mukhopadhyay, N. K., Kim, J., You, S., Morello, M., Hager, M. H., Huang, W. C., Ramachandran, A., Yang, J., Cinar, B., Rubin, M. A. et al. (2014). Scaffold attachment factor B1 regulates the androgen receptor in concert with the growth inhibitory kinase MST1 and the methyltransferase EZH2. *Oncogene* 33, 3235-3245. 10.1038/onc.2013.294 23893242 PMC3934948

[BIO059955C33] O'Carroll, D., Erhardt, S., Pagani, M., Barton, S. C., Surani, M. A. and Jenuwein, T. (2001). The polycomb-group gene Ezh2 is required for early mouse development. *Mol. Cell. Biol.* 21, 4330-4336. 10.1128/MCB.21.13.4330-4336.2001 11390661 PMC87093

[BIO059955C34] Okamoto, I., Otte, A. P., Allis, C. D., Reinberg, D. and Heard, E. (2004). Epigenetic dynamics of imprinted X inactivation during early mouse development. *Science* 303, 644-649. 10.1126/science.1092727 14671313

[BIO059955C35] Oksuz, O., Narendra, V., Lee, C. H., Descostes, N., Leroy, G., Raviram, R., Blumenberg, L., Karch, K., Rocha, P. P., Garcia, B. A. et al. (2018). Capturing the onset of PRC2-mediated repressive domain formation. *Mol. Cell* 70, 1149-1162.e5. 10.1016/j.molcel.2018.05.023 29932905 PMC7700016

[BIO059955C36] Perez-Riverol, Y., Bai, J., Bandla, C., Garcia-Seisdedos, D., Hewapathirana, S., Kamatchinathan, S., Kundu, D. J., Prakash, A., Frericks-Zipper, A., Eisenacher, M. et al. (2022). The PRIDE database resources in 2022: a hub for mass spectrometry-based proteomics evidences. *Nucleic Acids Res.* 50, D543-D552. 10.1093/nar/gkab1038 34723319 PMC8728295

[BIO059955C37] Pintacuda, G., Wei, G., Roustan, C., Kirmizitas, B. A., Solcan, N., Cerase, A., Castello, A., Mohammed, S., Moindrot, B., Nesterova, T. B. et al. (2017). hnRNPK recruits PCGF3/5-PRC1 to the Xist RNA B-repeat to establish polycomb-mediated chromosomal silencing. *Mol. Cell* 68, 955-969.e10. 10.1016/j.molcel.2017.11.013 29220657 PMC5735038

[BIO059955C38] Plath, K., Fang, J., Mlynarczyk-Evans, S. K., Cao, R., Worringer, K. A., Wang, H., De La Cruz, C. C., Otte, A. P., Panning, B. and Zhang, Y. (2003). Role of histone H3 lysine 27 methylation in X inactivation. *Science* 300, 131-135. 10.1126/science.1084274 12649488

[BIO059955C39] Raab, J. R., Smith, K. N., Spear, C. C., Manner, C. J., Calabrese, J. M. and Magnuson, T. (2019). SWI/SNF remains localized to chromatin in the presence of SCHLAP1. *Nat. Genet.* 51, 26-29. 10.1038/s41588-018-0272-z 30510238 PMC6339527

[BIO059955C40] Rank, L., Herring, L. E. and Braunstein, M. (2021). Evidence for the mycobacterial Mce4 transporter being a multiprotein complex. *J. Bacteriol.* 203, e00685-20. 10.1128/JB.00685-20 33649150 PMC8088607

[BIO059955C41] Rosenberg, M., Blum, R., Kesner, B., Aeby, E., Garant, J. M., Szanto, A. and Lee, J. T. (2021). Motif-driven interactions between RNA and PRC2 are rheostats that regulate transcription elongation. *Nat. Struct. Mol. Biol.* 28, 103-117. 10.1038/s41594-020-00535-9 33398172 PMC8050941

[BIO059955C42] Schertzer, M. D., Braceros, K. C. A., Starmer, J., Cherney, R. E., Lee, D. M., Salazar, G., Justice, M., Bischoff, S. R., Cowley, D. O., Ariel, P. et al. (2019a). lncRNA-induced spread of polycomb controlled by genome architecture, RNA abundance, and CpG Island DNA. *Mol. Cell* 75, 523-537.e10. 10.1016/j.molcel.2019.05.028 31256989 PMC6688959

[BIO059955C43] Schertzer, M. D., Thulson, E., Braceros, K. C. A., Lee, D. M., Hinkle, E. R., Murphy, R. M., Kim, S. O., Vitucci, E. C. M. and Calabrese, J. M. (2019b). A piggyBac-based toolkit for inducible genome editing in mammalian cells. *RNA* 25, 1047-1058. 10.1261/rna.068932.118 31101683 PMC6633203

[BIO059955C44] Schumacher, A., Faust, C. and Magnuson, T. (1996). Positional cloning of a global regulator of anterior-posterior patterning in mice. *Nature* 384, 648. 10.1038/384648a0 8984348

[BIO059955C45] Silva, J., Mak, W., Zvetkova, I., Appanah, R., Nesterova, T. B., Webster, Z., Peters, A. H., Jenuwein, T., Otte, A. P. and Brockdorff, N. (2003). Establishment of histone h3 methylation on the inactive X chromosome requires transient recruitment of Eed-Enx1 polycomb group complexes. *Dev. Cell* 4, 481-495. 10.1016/S1534-5807(03)00068-6 12689588

[BIO059955C46] R Core Team. (2023). R: A language and environment for statistical computing. R Foundation for Statistical Computing, Vienna, Austria.

[BIO059955C47] Townson, S. M., Kang, K., Lee, A. V. and Oesterreich, S. (2004). Structure-function analysis of the estrogen receptor alpha corepressor scaffold attachment factor-B1: identification of a potent transcriptional repression domain. *J. Biol. Chem.* 279, 26074-26081. 10.1074/jbc.M313726200 15066997

[BIO059955C48] Trotman, J. B., Lee, D. M., Cherney, R. E., Kim, S. O., Inoue, K., Schertzer, M. D., Bischoff, S. R., Cowley, D. O. and Calabrese, J. M. (2020). Elements at the 5’ end of Xist harbor SPEN-independent transcriptional antiterminator activity. *Nucleic Acids Res.* 48, 10500-10517. 10.1093/nar/gkaa789 32986830 PMC7544216

[BIO059955C49] Trotman, J. B., Braceros, K. C. A., Cherney, R. E., Murvin, M. M. and Calabrese, J. M. (2021). The control of polycomb repressive complexes by long noncoding RNAs. *Wiley Interdiscip Rev RNA* 12, e1657. 10.1002/wrna.1657 33861025 PMC8500928

[BIO059955C50] Tyanova, S., Temu, T., Sinitcyn, P., Carlson, A., Hein, M. Y., Geiger, T., Mann, M. and Cox, J. (2016). The Perseus computational platform for comprehensive analysis of (prote)omics data. *Nat. Methods* 13, 731-740. 10.1038/nmeth.3901 27348712

[BIO059955C51] Wang, J., Mager, J., Chen, Y., Schneider, E., Cross, J. C., Nagy, A. and Magnuson, T. (2001). Imprinted X inactivation maintained by a mouse Polycomb group gene. *Nat. Genet.* 28, 371-375. 10.1038/ng574 11479595

[BIO059955C52] Wang, X., Paucek, R. D., Gooding, A. R., Brown, Z. Z., Ge, E. J., Muir, T. W. and Cech, T. R. (2017). Molecular analysis of PRC2 recruitment to DNA in chromatin and its inhibition by RNA. *Nat. Struct. Mol. Biol.* 24, 1028-1038. 10.1038/nsmb.3487 29058709 PMC5771497

[BIO059955C53] Wei, C., Xiao, R., Chen, L., Cui, H., Zhou, Y., Xue, Y., Hu, J., Zhou, B., Tsutsui, T., Qiu, J. et al. (2016). RBFox2 binds nascent RNA to globally regulate polycomb complex 2 targeting in mammalian genomes. *Mol. Cell* 62, 875-889. 10.1016/j.molcel.2016.04.013 27211866 PMC5515591

[BIO059955C54] Yu, J.-R., Lee, C.-H., Oksuz, O., Stafford, J. M. and Reinberg, D. (2019). PRC2 is high maintenance. *Genes Dev.* 33, 903-935. 10.1101/gad.325050.119 31123062 PMC6672058

[BIO059955C55] Yu, B., Qi, Y., Li, R., Shi, Q., Satpathy, A. T. and Chang, H. Y. (2021). B cell-specific XIST complex enforces X-inactivation and restrains atypical B cells. *Cell* 184, 1790-1803.e17. 10.1016/j.cell.2021.02.015 33735607 PMC9196326

[BIO059955C56] Zhang, Y., Liu, T., Meyer, C. A., Eeckhoute, J., Johnson, D. S., Bernstein, B. E., Nusbaum, C., Myers, R. M., Brown, M., Li, W. et al. (2008). Model-based analysis of ChIP-Seq (MACS). *Genome Biol.* 9, R137. 10.1186/gb-2008-9-9-r137 18798982 PMC2592715

[BIO059955C57] Zhang, Q., Mckenzie, N. J., Warneford-Thomson, R., Gail, E. H., Flanigan, S. F., Owen, B. M., Lauman, R., Levina, V., Garcia, B. A., Schittenhelm, R. B. et al. (2019). RNA exploits an exposed regulatory site to inhibit the enzymatic activity of PRC2. *Nat. Struct. Mol. Biol.* 26, 237-247. 10.1038/s41594-019-0197-y 30833789 PMC6736635

[BIO059955C58] Zylicz, J. J., Bousard, A., Žumer, K., Dossin, F., Mohammad, E., Da Rocha, S. T., Schwalb, B., Syx, L., Dingli, F., Loew, D. et al. (2019). The implication of early chromatin changes in X chromosome inactivation. *Cell* 176, 182-197.e123. 10.1016/j.cell.2018.11.041 30595450 PMC6333919

