# Supplementary material for: A monoclonal antibody raised against human EZH2 cross-reacts with the RNA-binding protein SAFB
Source: Biol Open. 2023 Jun 7;12(6):bio059955. doi: 10.1242/bio.059955 (PMC10259849; doi:10.1242/bio.059955)
Supplement: Supplementary information [file biolopen-12-059955-s1.pdf]

Table S1.

Click here to download Table S1

Table S2.

Click here to download Table S2

Table S3.

Click here to download Table S3

Table S4.

Click here to download Table S4

Table S5.

Click here to download Table S5
